# Phytochemical Profiling and Larvicidal Activity of Ethanolic Extracts from *Persea americana* Mill. (Var. Lorena) Against *Aedes aegypti*

**DOI:** 10.3390/insects17010034

**Published:** 2025-12-25

**Authors:** Clara Barragán-Avilez, Paula Pareja-Loaiza, Katherine Girón Domínguez, Beatriz López-Monroy, Adriana E. Flores, Martha Sánchez-Bolívar, Jaime Luna-Carrascal, Leonardo C. Pacheco-Londoño, Nataly J. Galán-Freyle, Elkin Navarro Quiroz, Karina Castellanos-Romero, Ronald Maestre-Serrano, Roger Valle-Molinares, Fabián Espitia-Almeida

**Affiliations:** 1 Centro de Investigaciones en Ciencias de la Vida, Facultad de Ciencias Básicas y Biomedicas, Universidad Simón Bolívar, Barranquilla 080002, Colombia; clara.barragan@unisimon.edu.co (C.B.-A.); jaime.luna@unisimon.edu.co (J.L.-C.); leonardo.pacheco@unisimon.edu.co (L.C.P.-L.); nataly.galan@unisimon.edu.co (N.J.G.-F.); elkin.navarro@unisimon.edu.co (E.N.Q.); 2 Centro de Investigaciones en Ciencias de la Vida, Facultad de Ciencias de la Salud, Universidad Simón Bolívar, Barranquilla 080002, Colombia; paula.pareja@unisimon.edu.co; 3 Facultad de Ciencias de la Salud, Universidad Libre, Sectional Barranquilla, Puerto Colombia 081001, Colombia; katherinem.girond@unilibre.edu.co (K.G.D.); martham.sanchezb@unilibre.edu.co (M.S.-B.); ronaldy.maestres@unilibre.edu.co (R.M.-S.); 4 Universidad Autónoma de Nuevo León, Facultad de Ciencias Biológicas, San Nicolás de los Garza 66455, Mexico; beatriz.lopezmr@uanl.edu.mx (B.L.-M.); adriana.floressr@uanl.edu.mx (A.E.F.); 5 Facultad de Ciencias Básicas, Programa de Biología, Universidad del Atlántico, Puerto Colombia 081001, Colombia; karinacastellanos@mail.uniatlantico.edu.co (K.C.-R.); rogervalle@mail.uniatlantico.edu.co (R.V.-M.)

**Keywords:** *Aedes aegypti*, ethanolic extract, larvicidal activity, *Persea americana*, phytochemistry screening, UV-Vis and UHPLC

## Abstract

Dengue is a mosquito-borne viral disease transmitted by *Aedes aegypti*, the main vector in the Americas. The lack of effective antiviral treatments, limited vaccine coverage, and the increasing resistance of mosquitoes to conventional insecticides emphasize the need for alternative vector control strategies. Plant-derived larvicides represent a promising and eco-friendly approach. This study characterized the phytochemical profile of *Persea americana* Mill. (var. Lorena) and evaluated its larvicidal activity against *Ae. aegypti* (Rockefeller strain). The phytochemical profile was assessed through qualitative screening, UV-Vis spectrophotometry, and UHPLC analysis. Larvicidal activity was evaluated against third-instar larvae of *Ae. aegypti* (Rockefeller strain) and the median lethal concentration (LC_50_) values were determined. Preliminary screening of ethanolic extracts revealed the presence of various secondary metabolites of pharmacological relevance, including alkaloids, coumarins, tannins, flavonoids, saponins, triterpenes/sterols, and quinones. UV-Vis spectra displayed distinct absorption patterns, with a prominent peak near 260 nm, consistent with the presence of aromatic compounds. UHPLC profiling revealed high chemical diversity across different plant parts, with 70, 98, 71, and 52 peaks (above 1 × 10^5^ intensity) detected in seed, flower, pulp, and leaf extracts, respectively. Larvicidal bioassays showed significant activity, particularly in the seed extract, with LC_50_ values (µg/mL) of 3.8 (3.3–4.1) for seeds, 22.4 (21.8–23.9) for flowers, 23.0 (21.5–24.6) for pulp, and 29.7 (28.1–31.2) for leaves. This study highlights the larvicidal potential of ethanolic extracts from *P. americana* (var. Lorena), with the seed extract exhibiting the highest chemical diversity and bioactivity against *Ae. aegypti* larvae. The detection of key secondary metabolites, including flavonoids, alkaloids, and saponins, supports the development of an effective, plant-based larvicide for sustainable vector control strategies.

## Simple Summary

Our study evaluated whether extracts obtained from the seeds, leaves, bark, fruit, peel of the fruit, root, and flowers of *P. americana* (var. Lorena) were lethal to *Ae. aegypti* larvae. Additionally, we determined the preliminary phytochemical, UV-Vis, and HPLC profiles of each extract. We found that the extracts differ in the richness and abundance of metabolites. These differences in phytochemical profiles are essential to explaining the extracts’ varying potencies against *Ae. aegypti* larvae. Notably, the seed extract showed the highest potency and is emerging as a promising ingredient for developing a biopesticide against this mosquito.

## 1. Introduction

Dengue is one of the most important mosquito-borne viral diseases, with *Aedes aegypti* as the primary vector. Its global incidence has increased thirtyfold over the past 50 years, making it a serious public health threat, particularly in tropical and subtropical regions where environmental conditions favor mosquito proliferation [1,2]. Dengue is currently endemic in more than 100 countries, and approximately 70% of cases occur in Africa, the Americas, the Eastern Mediterranean, Southeast Asia, and the Western Pacific. These regions have experienced increasing morbidity and mortality, with an estimated 100–400 million infections per year and epidemic cycles every 3–5 years, influenced by climatic variability and human mobility [1,3,4,5].

According to the Pan American Health Organization, dengue continues to pose a major public health challenge in the region due to the widespread distribution of *Ae. aegypti* and the co-circulation of all four dengue virus serotypes (DENV-1 to DENV-4). In 2024, over 13 million cases were reported in the Americas, including 22,684 severe cases and 8186 deaths. By epidemiological week 4 of 2025, 355,621 probable cases had been recorded, corresponding to a cumulative incidence of 35 cases per 100,000 inhabitants, remaining above the five-year average despite a decrease compared to 2024 [6].

Efforts to prevent and control dengue face persistent challenges, including the re-emergence of outbreaks, limitations in sanitation and urban planning, the lack of broadly effective vaccines, and increasing insecticide resistance in *Ae. aegypti* populations [7,8]. These constraints underscore the need to investigate complementary tools within integrated vector management. Among such alternatives, plant-derived products have attracted growing interest due to their chemical diversity and potential biological activity against mosquito vectors [9,10]. Their use has also been proposed as an environmentally compatible approach that may reduce reliance on synthetic insecticides; however, their safety and efficacy must be established experimentally and cannot be assumed a priori.

*Persea americana* Mill. (avocado), a member of the Lauraceae family, is widely cultivated in tropical and subtropical regions and contains diverse bioactive metabolites that have prompted interest in various industrial and research fields [11,12]. Different parts of the plant, including fruit, seeds, stem, bark, and leaves, have been traditionally used in folk medicine and have been reported to exhibit antiviral, antioxidant, antimicrobial, and insecticidal properties [13,14,15,16,17,18,19]. Studies from Brazil, Cuba, the Philippines, Mexico, and Venezuela describe larvicidal activity of essential oils from local *P. americana* varieties against *Ae. aegypti* [20,21,22,23]. Although some reports indicate promising activity, the chemical complexity of essential oils requires careful evaluation, as their composition, efficacy, and safety may vary substantially among cultivars and extraction methods.

Given the influence of geographical and genetic variation on metabolite profiles, exploring locally grown cultivars is essential. In Colombia, the ‘Lorena’ variety of *P. americana* is widely cultivated and economically relevant; however, its larvicidal potential has not been previously evaluated, and therefore no assumptions can be made regarding its biological activity or safety.

This study aimed to assess the larvicidal activity of *P. americana* var. Lorena collected in the Montes de María region (Department of Bolívar, northern Colombia). Evaluating native plant materials contributes to identifying potential candidates for future vector control research, while generating evidence-based information on their efficacy and possible applications.

## 2. Materials and Methods

### 2.1. Study Area and Collection of Plant Material

Plant material of *P. americana* (var. Lorena) was collected in June 2023 from a smallholder-managed agroforestry plantation located in the community of El Camarón (9°50′26.36″ N, 75°17′2.57″ W; altitude: 154.5 m a.s.l.), located in Carmen de Bolívar, Bolívar Department, Colombia. Plant parts were manually harvested directly from the tree and placed in paper bags for transport to the Chemistry and Nanotechnology Laboratory at Universidad Simón Bolívar.

Upon arrival, the plant material was separated into leaves, bark, roots, flowers, seeds, pulp, and fruit peel. The fruit peels were washed with distilled water to remove surface contaminants. All plant material was then placed on clean steel trays lined with kraft paper and dried in a ventilated oven at 40 °C for seven days.

### 2.2. Preparation of Extracts

Dried plant material was ground using an industrial food grinder. A total of 50 g of each pulverized plant part was weighed and macerated in amber glass bottles with 500 mL of ethanol at 96% (1:10 *w*/*v*) at room temperature for four weeks. After the extraction period, the mixtures were filtered using Qualitative filter paper, grade 1 (Whatman No. 1; Cytiva, Marlborough, MA, USA) and concentrated under reduced pressure using a rotary evaporator (Halthen High Precision MMRE-04, Shanghai, China) at 45 °C. The dried extracts were then weighed and stored in glass vials at 4 °C, protected from light until further use [24].

### 2.3. Preliminary Phytochemical Screening

Preliminary phytochemical screening of the ethanolic extracts of *P. americana* (var. Lorena) was conducted using two different assays for the identification of each group of secondary metabolites. The procedures followed previously published protocols, adapted and standardized by our research group [25,26]. The ethanolic extracts were analyzed to detect the presence of alkaloids (Dragendorff’s and Wagner’s assays), tannins (ferric chloride and gelatin salt assays), coumarins (Bornträger’s and fluorescence assays), flavonoids (Shinoda and citroboric assays), triterpenes/sterols (Liebermann-Buchard and Salkowski assays), saponins (foam and α-naphthol assays), and quinones (Bornträger’s and Meyer’s assays).

Phytochemical plate assays were carried out using thin-layer chromatography (TLC) on silica gel 60 F254 plates (Merck, Darmstadt, Germany), with an eluent composed of chloroform/ethyl acetate (7:3 *v*/*v*) (solvents from Merck, Hohenbrunn, Germany). Tube assays were performed in 13 mL capped glass tubes.

The results of the phytochemical screening are expressed as relative concentrations of metabolites, using the following scale: abundant [++], low [+], and not detected [-].

### 2.4. UV-Vis Analysis

To measure the UV-Vis spectra, a stock solution was prepared by weighing 5 mg of each extract obtained from the plant parts (leaves, bark, roots, flowers, seeds, pulp, and fruit peel) and dissolving it in 5 mL of reagent-grade ethanol. The solutions were vortexed (LP Vortex Mixer, Thermo Scientific, Seoul, Republic of Korea) and sonicated (Elmasonic E60H, Singen, Germany) for 10 min. Each extract was then filtered using Whatman No. 1 filter paper (MA, USA).

For the qualitative determination of the UV-Vis fingerprint profile, 1:20 dilutions of the stock solutions were prepared in ethanol. These working solutions were transferred to 2 mL quartz cuvettes and scanned over the wavelength range of 190–1100 nm using a spectrophotometer (Genesys 150, Thermo Scientific Genesys, Madison, WI, USA).

### 2.5. UHPLC Analysis

Each of the seven extracts (leaves, bark, roots, flowers, seeds, pulp, and peel) was dissolved in 200 µL of cold methanol, vortexed (LP Vortex Mixer, Thermo Scientific, Republic of Korea), and sonicated in an ultrasonic bath (Elmasonic E60H, Singen, Germany) for 10 min. The samples were then incubated on ice for 1 h and centrifuged at 10,000× *g* for 10 min. Subsequently, 5 µL of the supernatant from each sample was transferred to UHPLC vials and placed in the autosampler for chromatographic analysis under the specified conditions. Chromatographic separation was performed using a Kinetex C18 column (2.1 mm ×100, particle size 1.7 µm; Phenomenex, Torrance, CA, USA) maintained at 40 °C. The mobile phase consisted of solvent A (water with 0.1% formic acid) and solvent B (acetonitrile with 0.1% formic acid), delivered at a flow rate of 400 µL/min. The UV-VIS detector was set to a wavelength of 260 nm. The following gradient was applied: 0 min, 88% A; 12.5 min, 70% A; 14.5 min, 40% A; and 19 min, 88% A, with a total run time of 19 min. The UHPLC analysis was performed using an Ultra High-Performance Liquid Chromatography system (Bruker Daltonik GmbH, Bremen, Germany), and chromatographic data were processed using DataAnalysis software version 4.4.

### 2.6. Bioassays

#### 2.6.1. Biological Material

The larvae of *Ae. aegypti* (Rockefeller strain), a reference strain known for its susceptibility to insecticides, were obtained from the Centers for Disease Control and Prevention (CDC), Atlanta, GA, USA, and have been maintained at the Entomology Laboratory at Universidad Simón Bolívar since generation F55. The eggs were placed in trays with dechlorinated water until hatching, and the first-instar larvae were reared under controlled environmental conditions: temperature of 28 ± 2 °C, 60 ± 10% RH, and a 12:12 h light–dark photoperiod. The larvae were fed a 10% liver-based diet (powdered liver concentrate, 290037790, MP Biomedicals, Solon, OH, USA) until they reached the third instar. Third-instar larvae were distinguished according to established morphological diagnostic features, including cephalic capsule width, siphon development, and degree of body segmentation, following the identification criteria described by [27].

#### 2.6.2. Larvicidal Activity

To evaluate the larvicidal activity of the seven extracts, insect mortality was assessed following the WHO protocol [27]. A total of 100 third-instar larvae were used for each extract, divided into four replicates of 25 larvae, and exposed to a concentration of 200 µg/mL in 100 mL of dechlorinated water; this concentration was used for preliminary screening tests of larvicidal activity among multiple plant extracts. Extracts that produced measurable mortality at this concentration were subsequently evaluated in a full dose–response assay (1–200 µg/mL) to determine their LC_50_ values.

Temephos at the WHO-recommended diagnostic dose (0.012 ppm) was used as the positive control, while dimethyl sulfoxide (DMSO) at a concentration ˂ 1% served as a negative control. Larval mortality was recorded after 1, 3, 6, 9, 12, and 24 h of exposure. Each assay was performed in triplicate, for a total of 300 larvae per extract.

Larvae were considered dead if they did not respond to physical contact with a thin glass rod. Tests were considered invalid if more than 10% of the larvae in the negative control died or if more than 10% developed into pupae. The mortality percentage was calculated as the number of dead larvae relative to the total number exposed.

Extracts that caused >80% mortality after 24 h were selected for subsequent determination of median lethal concentration (LC_50_). Extracts that did not induce larval mortality at 200 µg/mL were considered inactive. Mortality rates were corrected relative to the control using the Abbott formula [28].

$$Adjusted\;mortality\;\left(\%\right)=\frac{\%\;Treatment\;mortality-\%\;Control\;mortality}{100-\%\;Control\;mortality}\times 100$$

#### 2.6.3. Median Lethal Concentration (LC_50_)

LC_50_ values were determined by probit analysis, based on concentration–response curves obtained after 24 h of exposure to a series of concentrations ranging from 1 to 200 μg/mL of the active extracts.

#### 2.6.4. Statistical Analysis

Data analysis and graphical representation were performed using GraphPad Prism software, version 9.3.0 (GraphPad, San Diego, CA, USA). The F-test based on the sum of squares was used for statistical comparisons of LC_50_ values, with a significance level set at α = 0.05.

## 3. Results

### 3.1. Phytochemical Screening

The preliminary phytochemical screening of ethanolic extracts of *P. americana* (var. Lorena) revealed the presence of seven families of bioactive compounds of pharmacological interest, including alkaloids, coumarins, tannins, flavonoids, saponins, triterpenes/sterols, and quinones, with varying levels of abundance.

Alkaloids were detected in the seeds, leaves, flowers, pulp, and fruit peel extracts, appearing as orange or yellow spots in the low-polarity zone after reaction with Dragendorff’s and Wagner’s reagents. Coumarins were identified in the seed, flower, bark, and root extracts by blue fluorescence under UV light (365 nm) following development with Bornträger’s reagent. Tannins were detected in all extracts by the appearance of blue or green coloration after reaction with 1% ferric chloride and gelatin salt reagents.

The presence of flavonoids was confirmed in all extracts through both chromatographic and colorimetric assays. When developed with citroboric reagent and observed under UV light (365 nm), red, yellow, and blue fluorescent zones were detected in the high-polarity region of the chromatograms. In addition, the Shinoda test produced color changes to orange, red, pink, blue, or violet, confirming the presence of flavonoids in all samples.

Saponins were detected in all extracts based on positive reactions with the foam test and the α-naphthol test. Triterpenes and sterols were identified in the seed, leaf, flower, bark, and pulp extracts using the Libermann–Burchard test, which produced a green color indicative of sterols and a pink color indicative of triterpenes. These results were confirmed with the Salkowski test, where a yellow coloration indicated the presence of triterpenes. Quinones were detected exclusively in the seed extract, evidenced by red or pink color changes after reaction with Bornträger’s and Meyer’s reagents.

In addition, the extraction yields percentual (%; *w*/*w*) for each plant part are presented in Table 1. Most extracts yielded >10%; however, the flower, fruit peel, and bark extracts showed lower yields of 8.8, 6.2, and 4.7%, respectively.

### 3.2. The UV-Vis Profile

The UV-Vis spectra of the seven ethanolic extracts of *P. americana* (var. Lorena) (Supplementary Figure S1) showed distinct absorption patterns, reflecting the diversity and relative abundance of phytochemical families present in each extract. Despite these differences, all extracts shared a common feature, a maximum absorption wavelength near 260 nm. This band is characteristic of aromatic compounds, including alkaloids, coumarins, flavonoids, and tannins, among others. Based on this observation, UHPLC analyses were performed using 260 nm as the detection wavelength.

Table 2 summarizes the absorption peak profiles for the UV-Vis spectra for each extract, expressed in terms of wavelength (nm) and absorbance. The seed extract showed the simplest profile, with only two high-intensity peaks, followed by the fruit peel extract with five peaks. In contrast, the most complex spectra were observed in the leaf extract (10 peaks), the peel extract (9 peaks), and the pulp extract (8 peaks).

### 3.3. UHPLC Profile

The qualitative analysis of the UHPLC profiles of the ethanolic extracts of *P. americana* (var. Lorena), recorded at 260 nm, revealed a broad diversity of compounds across different plant parts. The UHPLC of the seed, flower, pulp, and leaf extracts showed the highest chemical diversity, with 70, 98, 71, and 52 peaks, respectively. In contrast, the root, bark, and fruit peel extracts exhibited lower diversity, with 28, 51, and 49 peaks, respectively. The same extracts (seed, flower, pulp, and leaf) also displayed the peaks with the highest intensities in the chromatograms (Supplementary Figure S2). Using a threshold of 1 × 10^5^ in peak intensity to define relative abundance, these extracts were identified as the most enriched in detectable compounds. Table 3 presents the chromatographic parameters of peaks with intensity values above 1 × 10^5^ for each extract analyzed.

### 3.4. Larvicidal Screening and LC_50_ Determination

The seven ethanolic extracts of *P. americana* (var. Lorena) described in Table 1 were evaluated against third-instar larvae of *Ae. aegypti* (Rockefeller strain) at a screening concentration of 200 µg/mL to identify the most active extracts and determine their median lethal concentrations (LC_50_), (Supplementary Figure S3). At this screening concentration, the seed, flower, pulp, leaf, and root extracts exhibited high larvicidal activity, with more than 90% mortality observed after 24 h of exposure. In contrast, the bark and fruit peel extracts were inactive under the same conditions. In all assays, no mortality was observed in the control group (1% DMSO), whereas the positive control (temephos, 0.012 ppm) produced 100% mortality.

The LC_50_ values, along with their 95% confidence intervals, were as follows: 3.8 (3.3–4.1) µg/mL for seed, 22.4 (21.8–23.9) µg/mL for flower, 23.0 (21.5–24.6) µg/mL for pulp, 29.7 (28.1–31.2) µg/mL for leaf, and 49.4 (46.4–52.5) µg/mL) for root extracts. These data, along with the parameters of the fitted probit model (slope, χ^2^, and *p*-value), are presented in Table 4.

## 4. Discussion

Preliminary phytochemical analyses revealed the presence of seven main families of secondary metabolites: alkaloids, coumarins, tannins, flavonoids, saponins, triterpenes, sterols, and quinones. These results indicate that the Lorena variety of *P. americana* is rich in pharmacologically relevant metabolites, consistent with previous findings in other avocado varieties [11,14,29,30,31]. These compounds, known for their diverse biological and pharmacological properties, were differentially distributed among plant organs, highlighting the chemical diversity and complexity of the species. Notably, alkaloids were detected in several organs, including seeds, leaves, flowers, pulp, and fruit peel, suggesting multifunctional roles in plant physiology, possibly related to defense mechanisms or chemical signaling processes [32,33,34,35].

On the other hand, quinones showed a more restricted distribution, being detected exclusively in the seeds. This specific localization may be associated with a protective role during germination or with defense against predators and pathogens in early developmental stages [29,36,37]. These differences in the presence and concentration of secondary metabolites across plant organs demonstrate that each part of the plant has a unique phytochemical profile. This diversity not only carries ecological and functional significance but also offers potential for applications in the development of pharmaceuticals, nutraceuticals, and cosmetic products [38,39,40,41].

Spectroscopic analysis in the ultraviolet-visible (UV-Vis) region revealed that all the extracts exhibited a maximum absorption near 260 nm (Supplementary Figure S1). This wavelength is characteristic of the conjugated aromatic structures typically present in several classes of secondary metabolites, including alkaloids, coumarins, flavonoids, and tannins. Absorption in this region primarily results from π → π* electronic transitions within aromatic ring systems [42,43,44]. Although all extracts shared a similar absorption maximum, notable differences were observed in the spectral profiles, particularly in peak intensity and curve shape. These variations reflect both qualitative and quantitative differences in chemical composition, suggesting varying relative abundances and the possible presence of unique compounds in each extract. Therefore, UV-Vis analysis not only confirms the presence of aromatic metabolites but also provides valuable preliminary information on the chemical diversity and potential biological activity of each plant extract.

The chromatographic profiles obtained by UHPLC revealed a remarkable diversity of compounds across the different plant parts [30,42,43,45]. Notably, the seed, flower, pulp, and leaf extracts showed the highest phytochemical richness, with 70, 98, 71, and 52 peaks detected, respectively (Supplementary Figure S2). This high diversity suggests that these organs are metabolically active, potentially due to their specific roles in physiological and ecological functions such as response to abiotic and biotic stress and participation in growth and development [46,47]. In addition, the chromatograms of these extracts displayed the highest intensity peaks, further indicating a higher relative abundance of bioactive compounds, many of which may be involved in antioxidant, antimicrobial, or defense-related activities against herbivores and pathogens [36,37,48,49].

By contrast, extracts from the root, bark, and fruit peel exhibited lower chemical complexity, evidenced by both a reduced number of detected peaks and lower chromatographic signal intensity. This pattern suggests that these organs may contribute less to secondary metabolite accumulation, or that they are dominated by a limited number of specialized compounds with specific physiological functions.

The evaluation of larvicidal activity against *Ae. aegypti* revealed that seed, flower, pulp, leaf, and root extracts of *P. americana* induced >90% mortality at 200 µg/mL. LC_50_ showed that the seed extract exhibited the highest potency, with an LC_50_ of 3.8 µg/mL, followed by the flower, pulp, leaf, and root extracts, with LC_50_ values of 22.4 µg/mL, 23.0 µg/mL, 29.7 µg/mL, and 49.4 µg/mL, respectively (Table 4). These results highlight the potential of *P. americana* extracts, particularly the seed extract, as promising natural agents for vector control.

Overall, the results are consistent with previous studies. For instance, Torres et al. [23] evaluated a variety of *P. americana* cultivated in the Philippines and reported that extracts containing low- and medium-polarity metabolites exhibited toxicity against larvae of the *Ae. aegypti* Rockefeller strain. In their study, seed extracts also demonstrated higher potency, with an LC_50_ of 16.48 µg/mL and an LC_90_ of 45.77 µg/mL [23], similar to those obtained in our study, where the seed extract presented an LC_50_ of 3.8 (3.7–4.1) µg/mL (Table 4).

In line with these findings, Agrela et al. [20] evaluated a creole variety of *P. americana* collected in Venezuela and found that seed extracts exhibited high larvicidal activity against larvae of *Ae. aegypti* Rockefeller strain, reporting an LC_50_ 5.7 µg/mL, similar to that obtained in our study (Table 4). Similarly, Molina-Beltrán et al. [50] demonstrated that a creole avocado variety collected in Cuba had a larvicidal potential, with an LC_50_ of 17.4 µg/mL against larvae of *Ae. aegypti* Rockefeller strain. In Mexico, Ramos-Casillas et al. [51] assessed the Hass variety and reported an LC_50_ of 20.4 µg/mL, also confirming the activity of *P. americana* against mosquito larvae. By contrast, Serejo et al. [22] employed a hydroalcoholic extraction method (70% *v*/*v* ethanol) of *P. americana* seeds collected in Brazil and reported low larvicidal efficacy, with LC_50_ values ranging from 181.7 to 401.9 µg/mL against larvae of the *Ae. aegypti* Rockefeller strain. The differences observed in larvicidal activity among studies may be attributed to factors including the avocado variety, pedological conditions, harvest season, and geographical origin, all of which can influence the phytochemical composition and bioactivity.

Several compounds from *P. americana* have been identified for their potential biological activity. Ramos-Casillas et al. [51] reported a high content of triterpenes, sesquiterpene lactones, and fatty acids such as 1, 2, 4-trihydroxy-nonadecane and 1, 2, 4-trihydroxy-heptadecane in seed extracts, and attributed the larvicidal activity of the methanolic seed extract specifically to the presence of triterpenes and sesquiterpene lactones. Guillén-Andrade et al. [31] identified estragole as one of the most abundant compounds in *P. americana*, highlighting its antifungal, larvicidal, insecticidal, and genotoxic activity. Other bioactive compounds isolated from avocado include β-pinene, caryophyllene, hexadecanoic acid, and α-tocopherol heptacosanom. Additionally, Rodriguez-Saona et al. [52] isolated 2-pentadecylfuran and 2-heptadecylfuran from *P. americana*, both of which exhibited 100% larvicidal activity against Spodoptera exigua, a common pest of avocado crops.

According to previous studies, avocado seed extracts typically contain a variety of compounds, including alkaloids, coumarins, tannins, flavonoids, saponins, triterpenes, and sterols, which are believed to be responsible for their larvicidal activity. These compounds can interfere with the development and growth of insect larvae, ultimately leading to their death [31,51]. In our study, phytochemical screening revealed a significant presence of these compound families in the endosperm extract (Table 1), which could explain the high larvicidal activity against *Ae. aegypti* (Rockefeller strain). Notably, when comparing the larvicidal potency of seed extracts reported in the literature with the endosperm extract analyzed in this study, our extract exhibited higher larvicidal activity as evidenced by the lower LC_50_ value (Table 2 and Table 3).

The findings presented here demonstrate the high larvicidal potential of ethanolic extracts from the endosperm, leaves, episperm, and flowers of *P. americana* (var. Lorena) against *Ae. aegypti*. Among these, the endosperm extract is the most promising candidate for future studies aimed at isolating and characterizing the active fractions or pure metabolites responsible for the larvicidal effect, with the goal of developing a novel and effective larvicide for mosquito control. However, it is important to note that the results reported here were obtained using the Rockefeller strain, which is susceptible to insecticides. Therefore, future evaluations should include field-collected populations with varying levels of resistance to assess the real-world efficacy of these extracts. One of the challenges encountered during this research was limited access to certain plant collection sites, which may affect the scalability or broader application of the findings.

On the other hand, given that this study was conducted under controlled laboratory conditions, it is possible that our results cannot be fully replicated under environmental conditions due to the various ecological interactions present in natural *Ae. aegypti* breeding sites. Although temephos was included at the WHO-recommended diagnostic dose (0.012 ppm) as a positive control, its performance may differ under field conditions due to the well-documented variability associated with emerging resistance patterns in some mosquito populations. These factors highlight the need for further research into the chemistry of the extracts in order to identify and isolate the component that causes larval death, and to conduct field and semi-field trials to fully validate the operational potential of each extract and the stability of its components.

## 5. Conclusions

The comprehensive phytochemical, spectroscopic, chromatographic, and biological evaluation of ethanolic extracts from *P. americana* (var. Lorena) revealed a high diversity of bioactive secondary metabolites, differentially distributed across plant organs. Phytochemical screening confirmed the presence of alkaloids, coumarins, tannins, flavonoids, saponins, triterpenes/sterols, and quinones, with flavonoids, tannins, and saponins detected in all extracts, while quinones were restricted to seeds. UV-Vis spectroscopy showed consistent absorption maxima around 260 nm, characteristic of aromatic compounds, which guided the selection of this wavelength for UHPLC detection. UHPLC profiling revealed that seed, flower, pulp, and leaf extracts were the most chemically diverse and enriched, as indicated by the number and intensity of chromatographic peaks observed. These same extracts exhibited strong larvicidal activity against third-instar larvae of *Ae. aegypti* (Rockefeller strain), with the seed extract showing the highest potency (LC_50_ = 3.8 µg/mL). In contrast, bark and fruit peel extracts displayed lower chemical complexity and no significant larvicidal effect. Collectively, these findings indicate that the seed extract of *P. americana* (var. Lorena) is a particularly promising candidate for further development as a natural, plant-based larvicide. Future studies should include field-resistant mosquito populations to support their potential use in integrated vector control strategies.

## Figures and Tables

**Table 1:** Families of compounds of pharmacological interest identified in the extracts of *P. americana* (var. Lorena) during the preliminary phytochemical screening.

Metabolite	Test	Seeds	Flowers	Pulp	Leaves	Root	Bark	Fruit Peel
Alkaloids	D	++	++	++	++	-	-	++
	W	++	++	++	++	-	-	++
Coumarins	Bt	++	++	-	-	+	+	-
	F	++	++	-	-	+	+	-
Tannins	CF	++	+	++	++	++	+	+
	G-S	++	+	++	++	++	+	+
Flavonoids	Shi	++	+	+	+	+	+	+
	Cit	++	+	+	+	+	+	+
Saponins	T-E	++	+	+	++	+	+	+
	A-N	++	+	+	++	+	+	+
Triterpenes/Esterols	L-B	++	++	++	++	-	+	-
	S	++	++	++	++	-	+	-
Quinones	Bt	+	-	-	-	-	-	-
	Me	+	-	-	-	-	-	-
Extraction yield (% *w*/*w*)		16.4	8.8	12.7	20.5	11.5	6.2	4.7

D: Dragendorff; W: Wagner; Bt: Borntrager; F: fluorescence; CF: ferric chloride; G-S: gelatin–salt; Shi: Shinoda; Cit: citroboric; T-E: foam test; A-N: α-naphthol test; L-B: Lieberman–Burchard; S: Salkowski; Me: Meyer test. Metabolite content: low (+); abundant (++); not detected (-).

**Table 2:** UV-Vis absorption peak wavelengths (nm) and absorbance values of ethanolic extracts from different parts of *P. americana* (var. Lorena).

Bands	Extracts						
	Seeds	Flowers	Pulp	Leaves	Root	Bark	Fruit Peel
2	284 (1.45)	258 (1.23)	232 (0.63)	264 (1.18)	252 (1.14)	244 (0.52)	256 (1.34)
3		266 (1.25)	244 (0.72)	266 (1.17)	272 (1.18)	262 (1.50)	272 (1.38)
4		278 (1.12)	260 (1.28)	270 (1.15)	280 (1.34)	284 (1.45)	280 (1.45)
5		282 (1.10)	268 (1.25)	278 (1.09)	414 (0.07)	402 (0.38)	518 (0.06)
6		312 (0.69)	272 (1.24)	400 (0.35)	510 (0.04)	500 (0.03)	
7		414 (0.07)	282 (1.17)	502 (0.03)		544 (0.02)	
8			428 (0.01)	544 (0.02)		618 (0.02)	
9				602 (0.02)		668 (0.10)	
10				662 (0.09)			

**Table 3:** Retention times, integration areas, and true intensities of peaks greater than 1 × 10^5^.

Extract	Peak	RT (min)	Area	Intensity (10^5^)
Seeds	2	0.5	747,359	1.54
	18	7.1	1,768,170	3.37
	22	7.7	481,779	1.54
	24	7.9	275,551	1.06
	29	8.3	248,568	1.72
	30	8.4	1,312,707	2.45
	36	9.0	744,656	2.44
	38	9.2	566,583	2.46
	52	10.5	421,491	1.12
	56	11.0	679,891	2.90
	59	11.6	339,576	2.02
Flowers	5	0.8	821,363	1.06
	17	6.0	930,265	1.07
	21	6.6	505,343	1.05
	27	7.4	493,015	1.60
	28	7.5	652,578	2.10
	29	7.6	135,597	1.80
	32	8.2	180,459	2.0
	33	8.4	397,614	1.60
	35	8.6	428,385	1.40
	38	8.8	854,658	1.30
	39	9.0	988,646	2.30
	46	9.6	254,503	1.30
	47	9.7	411,742	1.80
	48	9.8	406,669	2.0
	49	9.9	775,668	2.30
	51	10.0	190,148	1.05
	52	10.1	354,853	1.50
	55	10.2	768,829	2.50
	69	11.3	314,755	1.30
	72	11.6	237,197	1.30
Pulp	2	0.5	640,142	3.32
	19	8.2	326,182	1.40
	22	8.4	246,246	1.13
	24	8.6	1,367,766	3.44
	28	8.9	313,610	1.42
	29	9.0	294,552	1.26
	35	9.6	452,148	2.14
	36	9.7	592,966	2.57
	37	9.8	847,903	4.08
	38	9.9	1,703,647	4.79
	39	10.0	548,805	2.98
	40	10.1	1,353,463	4.68
	41	10.2	454,132	1.59
	42	10.3	262,107	7.62
	43	10.4	973,921	3.79
	45	10.5	683,630	2.82
	56	11.3	877,422	4.01
Leaves	10	7.1	2,234,678	4.26
	13	7.9	413,512	1.04
	16	8.2	330,706	1.14
	17	8.4	2,027,402	3.21
	25	9.4	336,865	1.23
	26	9.5	568,168	1.76
Root	5	6.0	6,127,478	5.13
	6	6.3	3,730,287	3.27
	9	8.3	410,722	1.27
	25	12.7	752,736	2.09
Bark	32	9.1	385,659	1.42
	34	9.8	1,930,308	1.65
Fruit peel	2	0.5	790,867	2.89
	21	8.4	454,656	1.08

**Table 4:** Lethal concentrations (LC_50_) of ethanolic extracts of *P. americana* (var. Lorena) against third-instar *Ae. aegypti* (Rockefeller strain) larvae.

Extract	n	LC_50_ (µg/mL) (CI 95%)	Slope ± SD	*X*^2^ (df)	*p*-Value
Seeds	300	3.8 (3.7–4.1) a	5.21 ± 0.4	13.1 (6)	0.042
Flowers	300	22.4 (21.8–22.9) b	9.2 ± 1.2	2.6 (6)	0.034
Pulp	300	23.0 (21.5–24.6) b	11.3 ± 1.7	12.9 (6)	0.004
Leaves	300	29.7 (28.1–31.2) c	14.0 ± 1.6	19.2 (6)	0.042
Root	300	49.4 (46.4–52.5) d	1.9 ± 0.2	2.4 (6)	0.024
Bark	300	>200	--	--	--
Fruit peel	300	>200	--	--	--

n: number of larvae per treatment; LC_50_: median lethal concentration; CI 95%: 95% confidence interval; SD: standard deviation; df: degrees of freedom. Different letters in the LC_50_ column indicate significant differences based on non-overlapping 95% confidence intervals.

## Data Availability

The original contributions presented in this study are included in the article/Supplementary Material. Further inquiries can be directed to the corresponding author.

## Supplementary Materials

The following supporting information can be downloaded at: https://www.mdpi.com/article/10.3390/insects17010034/s1, Figure S1: UV-VIS profile of extracts obtained from *P. americana* (var. Lorena): seeds (a), flowers (b), pulp (c), leaves (d), root (e), bark (f), and fruit peel (g). Figure S2: UHPLC profiles of extracts from *P. americana* (var. Lorena): seeds (a), flowers (b), pulp (c), leaves (d), root (e), bark (f), and fruit peel (g). Figure S3: Concentration-response curves of ethanolic extracts of *P. americana* (Var. Lorena) against third instar larvae of *Ae. aegypti* (Rockefeller strain). Larval mortality was evaluated after 24 h of exposure to concentrations ranging from 1 to 200 µg/m. Panels represent extracts from seeds (A), flowers (B), pulp (C), leaves (D), and roots (E).
