# Supplementary material for: Phytochemical Profiling and Larvicidal Activity of Ethanolic Extracts from Persea americana Mill. (Var. Lorena) Against Aedes aegypti
Source: Insects. 2025 Dec 25;17(1):34. doi: 10.3390/insects17010034 (PMC12842180; doi:10.3390/insects17010034)
Supplement: Supplementary file 1 [file insects-17-00034-s001.zip › insects-3963954-supplementary.pdf]

Supporting Information

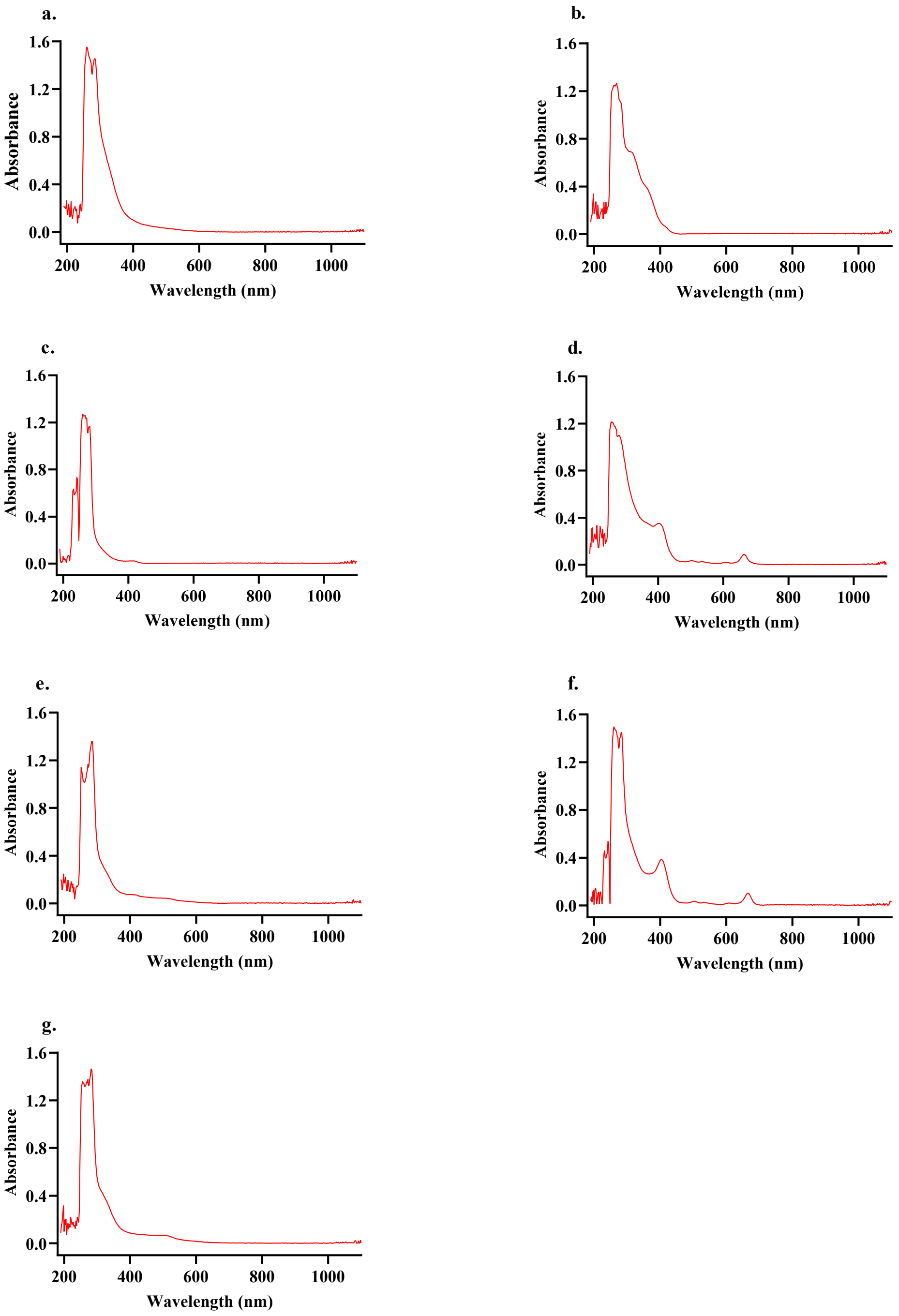

Figure S1. UV-VIS profile of extracts obtained from *P. americana* (var. Lorena): seeds (**a**), flowers (**b**), pulp (**c**), leaves (**d**), root (**e**), bark (**f**), and fruit peel (**g**).

a.

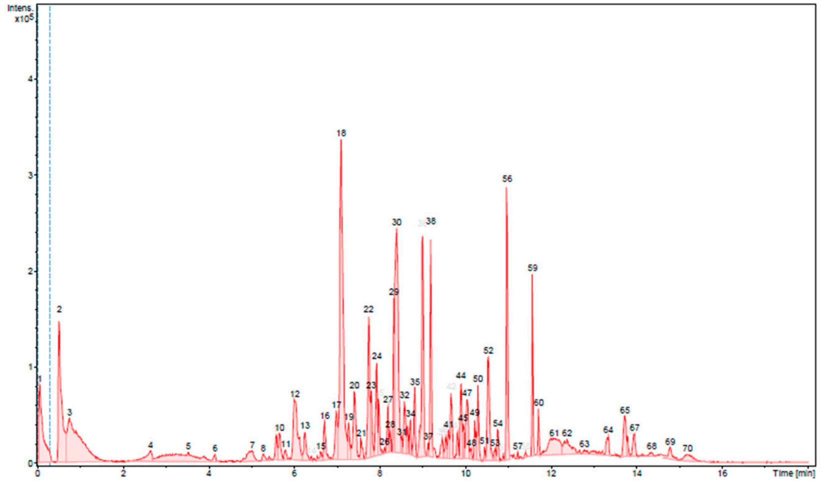
Intens.
x10^5
4
3
2
1
0
0
2
4
6
8
10
12
14
16
Time [min]

b.

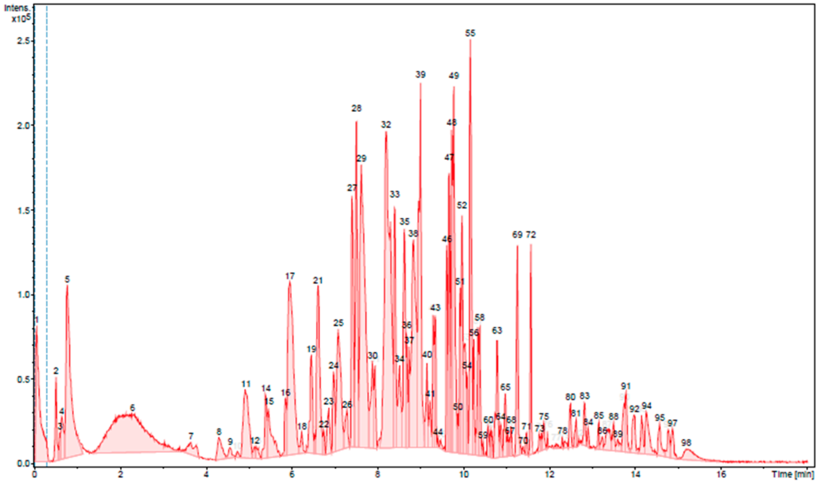
Intens.
x10^5
2.5
2.0
1.5
1.0
0.5
0.0
0
2
4
6
8
10
12
14
16
Time [min]

c.

d.

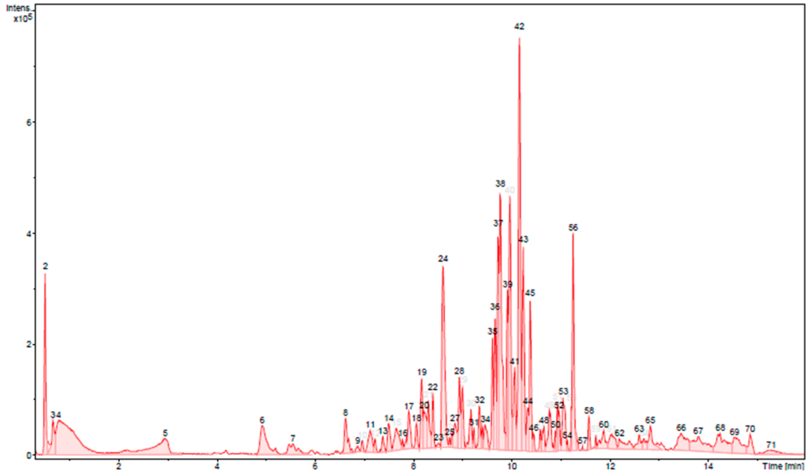
Intens.
x10^5
Time [min]

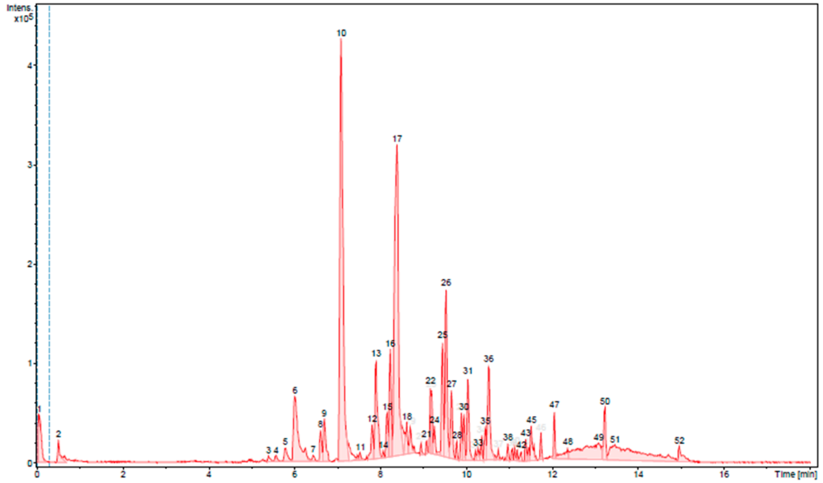
Intens.
x10^5
Time [min]

e.

f.

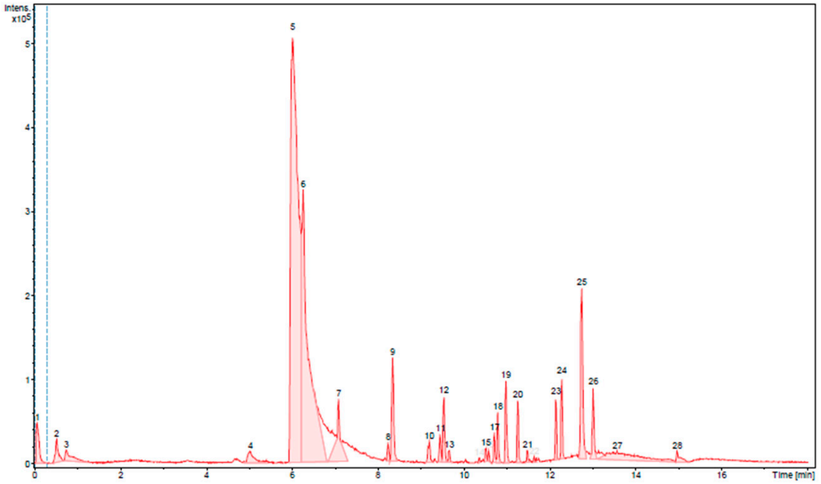
Intens.
x10^5
Time [min]

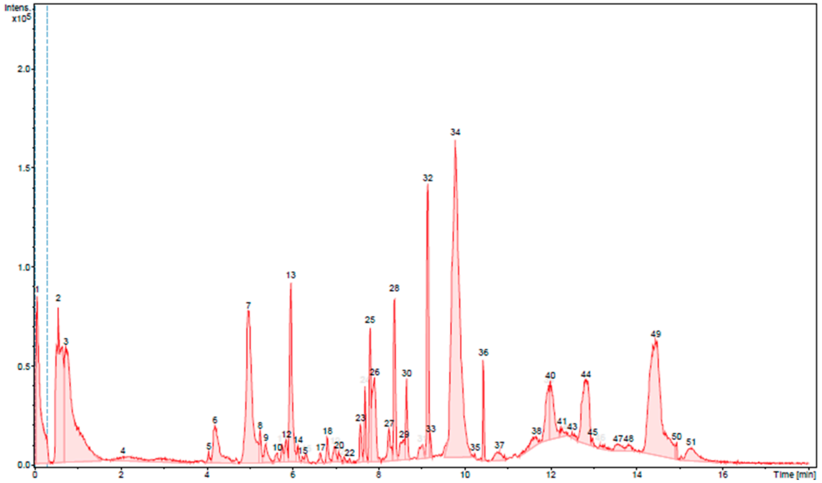
Intens.
x10^5
Time [min]

g.

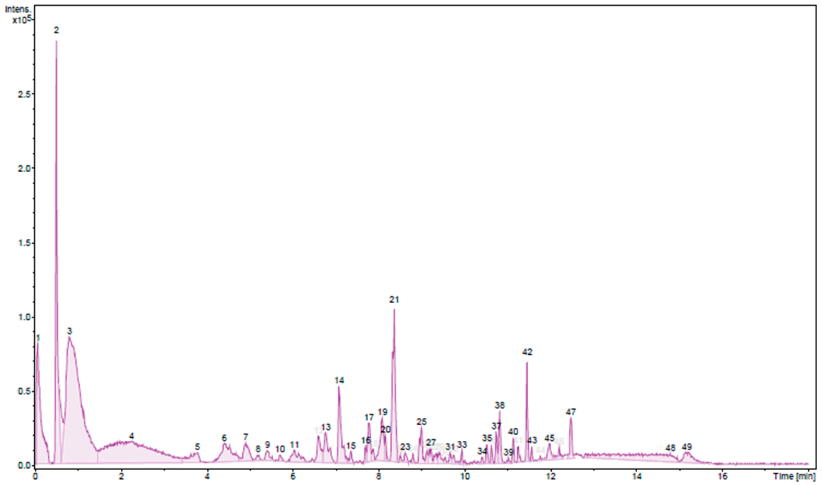

Figure S2. UHPLC profiles of extracts from *P. americana* (var. Lorena); seeds (**a**), flowers (**b**), pulp (**c**), leaves (**d**), root (**e**), bark (**f**) and fruit peel (**g**).

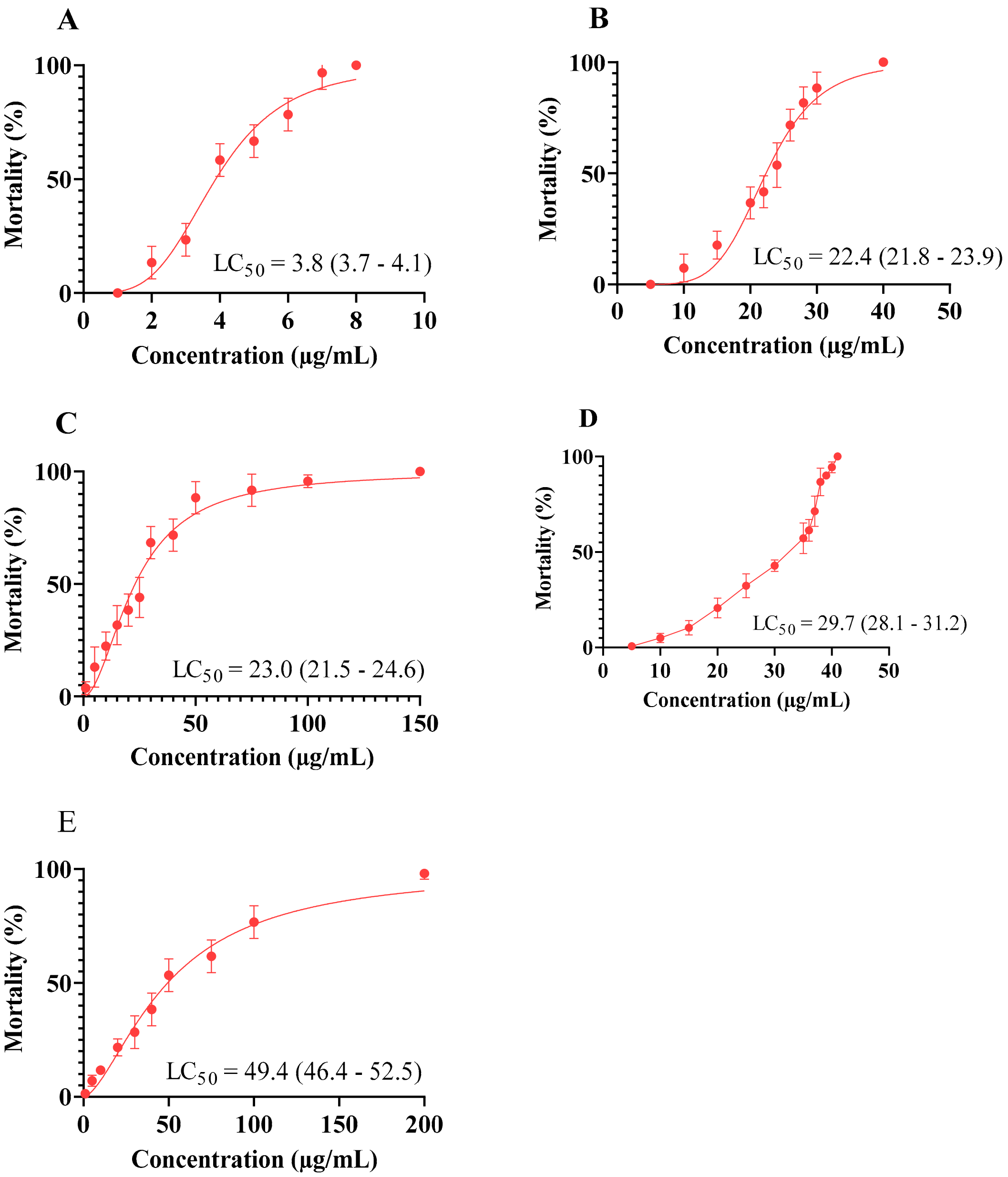

Figure S3. Concentration-response curves of ethanolic extracts of *P. americana* (Var. Lorena) against third instar larvae of *Ae. aegypti* (Rockefeller strain). Larval mortality was evaluated after 24 h of exposure to concentrations ranging from 1 to 200 µg/m. Panels represent extracts from seeds (A), flowers (B), pulp (C), leaves (D), and roots (E).
